# Editorial: Coronaviruses from the One Health perspective

**DOI:** 10.3389/fmicb.2023.1338529

**Published:** 2023-12-01

**Authors:** Maged Gomaa Hemida, Dilfuza Egamberdieva, Yashpal S. Malik

**Affiliations:** ^1^Department of Veterinary Biomedical Sciences, College of Veterinary Medicine, Long Island University, Brookville, NY, United States; ^2^Institute of Fundamental and Applied Research, National Research University TIAME, Tashkent, Uzbekistan; ^3^Medical School, Central Asian University, Tashkent, Uzbekistan; ^4^College of Animal Biotechnology, Guru Angad Dev Vety and Animal Sciences University, Ludhiana, Punjab, India

**Keywords:** One Health, coronaviruses, pandemic, emerging diseases, risk mitigation

Three coronaviruses have emerged in the past two decades [severe acute respiratory syndrome-1 (SARS-CoV-1), the Middle East respiratory coronavirus (MERS-CoV), and the SARS-CoV-2]. They represent ideal examples of the One Health concept (Hemida, 2019; Hemida and Ba Abduallah, 2020). This could be due to sharing common ancestors in bats and integrating some intermediate hosts as a reservoir for some of them. The dynamic changes in coronaviruses on the genomic level due to recombination and poor proof-reading capability of the coronavirus polymerases make possibilities for emerging new viruses/strains or even variants of the currently circulating strains. Minimizing the viral spread of some pathogens with pandemic potential from bats to intermediate host/vector will reduce the chance of any future potential pandemics.

Identifying the potential reservoir/es of SARS-CoV-2 is essential to prevent future infections and safeguard our food supply's security and stability. We approved and published four research articles for the current Research Topic. Al-Khalaifah et al. explored the relationship between avian CoV and relation with mammalian SARS-CoV2. The authors found that the avian and mammalian coronaviruses exhibit genetic and antigenic distinctions, making it improbable for poultry to serve as a vector for transmitting these coronaviruses. Fortunately, no substantiated evidence has indicated the potential cross-species transmission from human to bird despite the established transmission of SARS-CoV-2 from bats to humans.

The impact of bacterial coinfection with COVID-19 infection was assessed in the hospital. The study was the result of contradicting findings from the various studies that pointed out that the proportions of bacterial coinfection were very low (Garcia-Vidal et al., 2021) and the usage of antibiotics was very high in no apparent bacterial infection (Rawson et al., 2020). The neutrophil count and PCT level were used to distinguish the possibility of bacterial coinfection of COVID-19. The results showed that bacterial coinfection significantly raised the in-hospital mortality of COVID-19 patients (Zong et al.).

In another study, investigators established a link between COVID-19 respiratory illness and oropharyngeal microbiome severity. Authors have found that metabolic pathways associated with bacterial products may influence disease course positively (Bradley et al.). They are analyzing microbial abundance and the metabolic profiling of patients enhanced microbiome profiling's predictive potential for clinical outcomes. This underscores the oropharyngeal microbiome's potential diagnostic and therapeutic applications in combating respiratory viruses like SARS-CoV-2.

The persistence of SARS-CoV-2 and Pepper mild mottle virus (PMMoV) RNA in raw sewage from university dorms was investigated using real-time quantitative PCR (Li et al.). Results showed that the SARS-CoV-2 RNA decayed faster at 20°C than at 4°C and was detectable for at least 29 days, indicating evidence for the persistence of viral RNA in site-specific raw sewage water. This is particularly important for laboratories with limited resources and throughput, and sample storage is unavoidable. The study indicates an urgent need for attention to the examination of the decay of SARS-CoV-2 RNA at higher concentration levels and higher temperatures for a longer time.

The vigilant monitoring of common diseases with pandemic potentials, such as influenza and coronaviruses, is highly encouraged. Adaptation of the One Health-based control strategies would be among the critical measures to mitigate the risk of any potential future pandemics (Hemida and Alnaeem, 2019).

## Author contributions

MH: Conceptualization, Data curation, Formal analysis, Investigation, Methodology, Resources, Software, Validation, Visualization, Writing – original draft, Writing – review & editing. DE: Conceptualization, Data curation, Investigation, Methodology, Writing – original draft, Writing – review & editing. YM: Conceptualization, Formal analysis, Investigation, Methodology, Visualization, Writing – original draft, Writing – review & editing.
